# Supplementary material for: A 2D image 3D reconstruction function adaptive denoising algorithm
Source: PeerJ Comput Sci. 2023 Oct 3;9:e1604. doi: 10.7717/peerj-cs.1604 (PMC10557518; doi:10.7717/peerj-cs.1604)
Supplement: Supplemental Information 2 [file peerj-cs-09-1604-s002.zip › cs-85591-data/Effects/Figure_6.pdf]

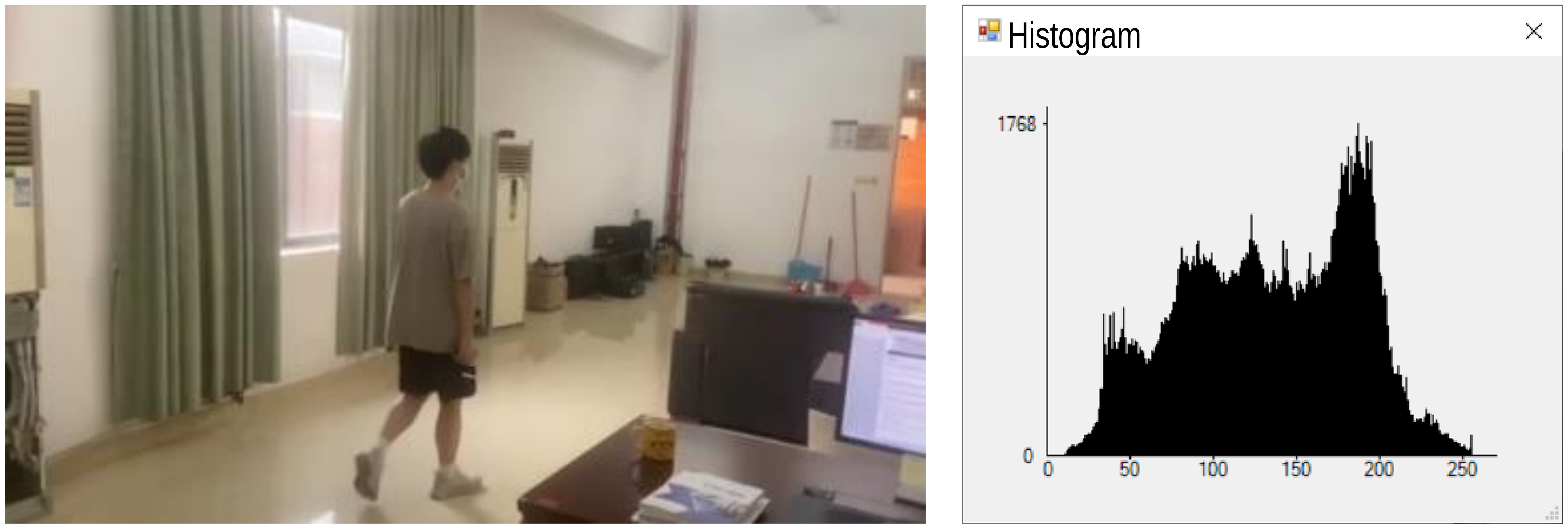

(a) Original image

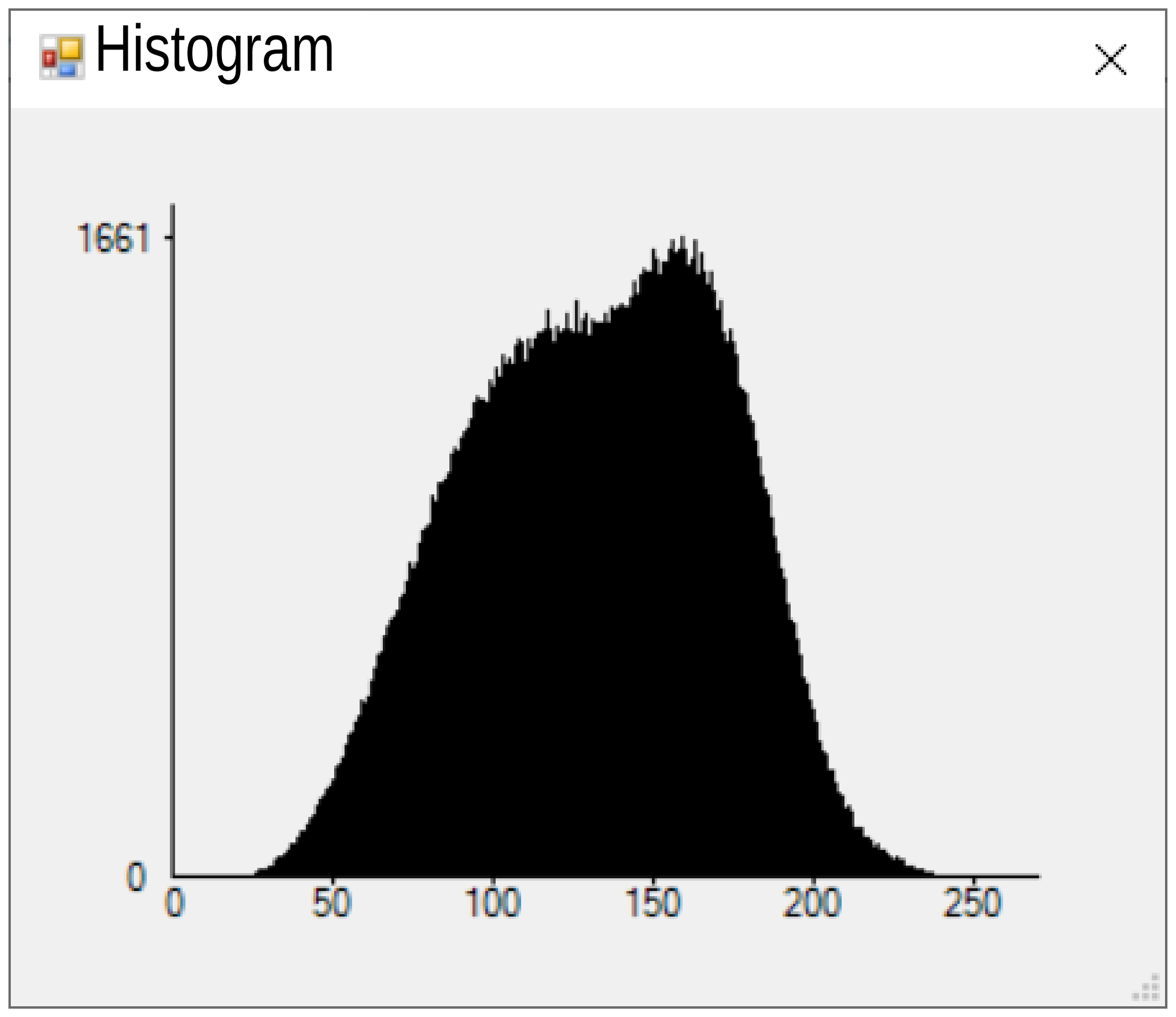

(b) Noisy image

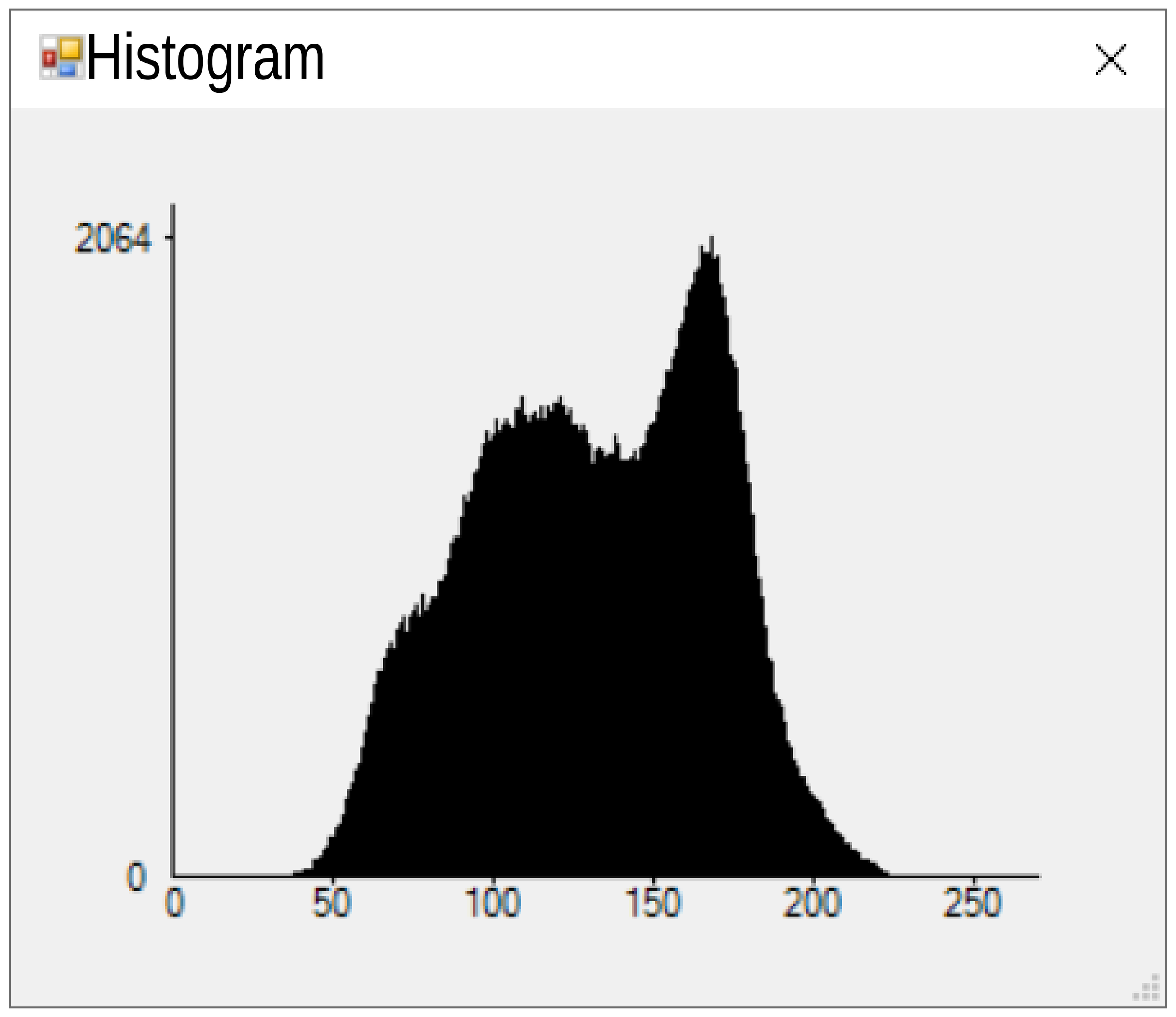

(c) Traditional Algorithm

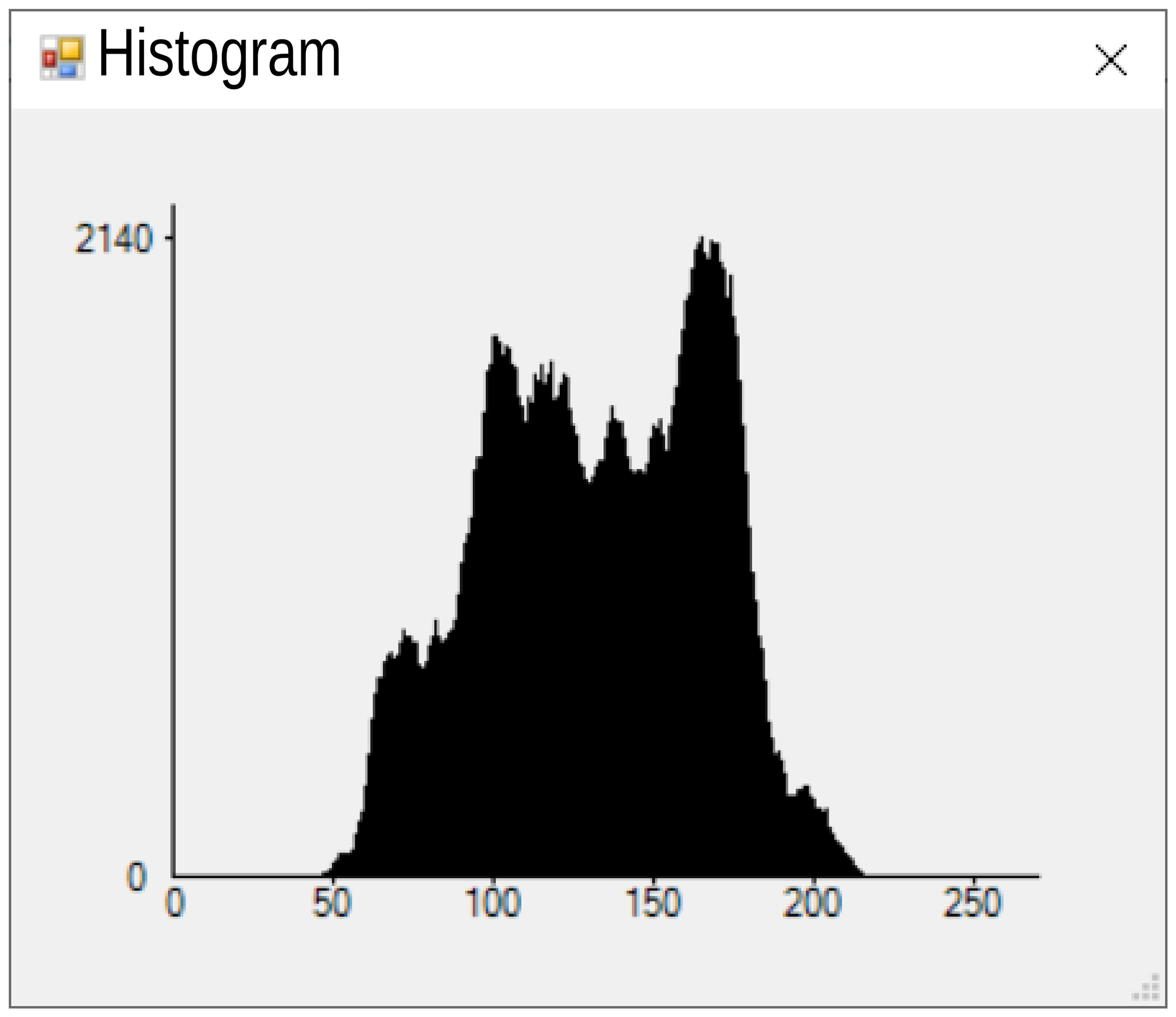

(d) Literature algorithm1

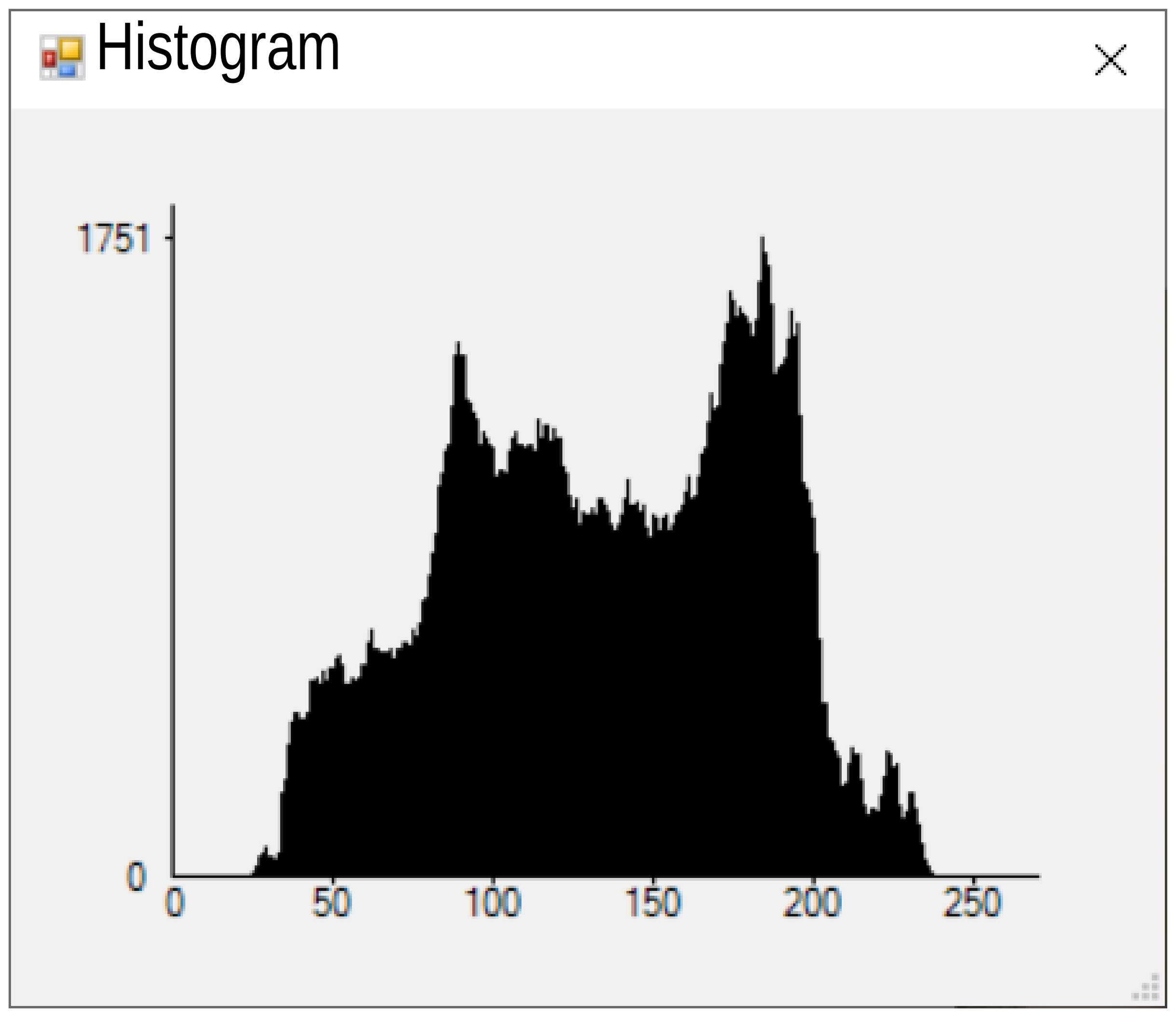

(e) Literature algorithm2

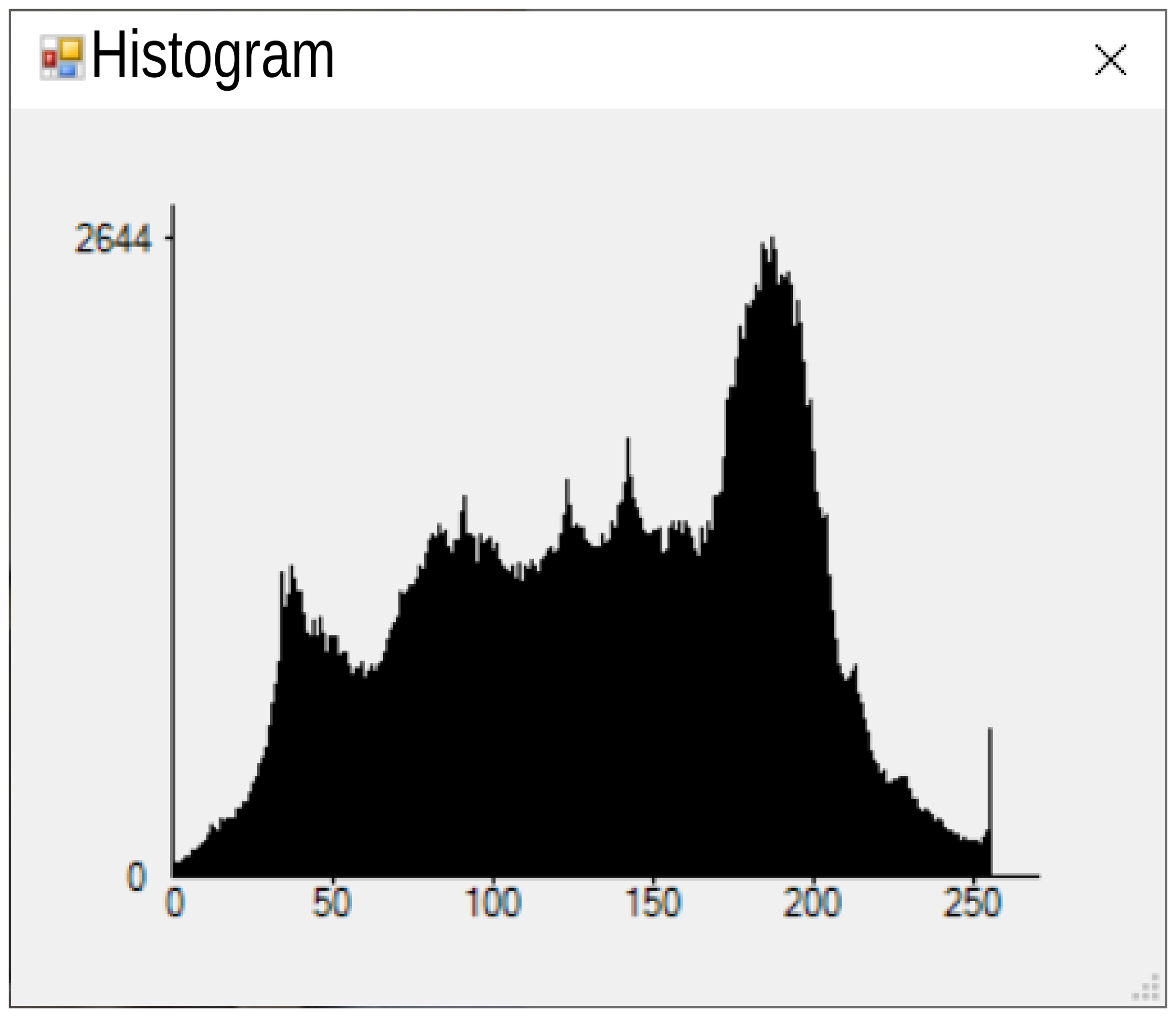

(f) Literature algorithm3

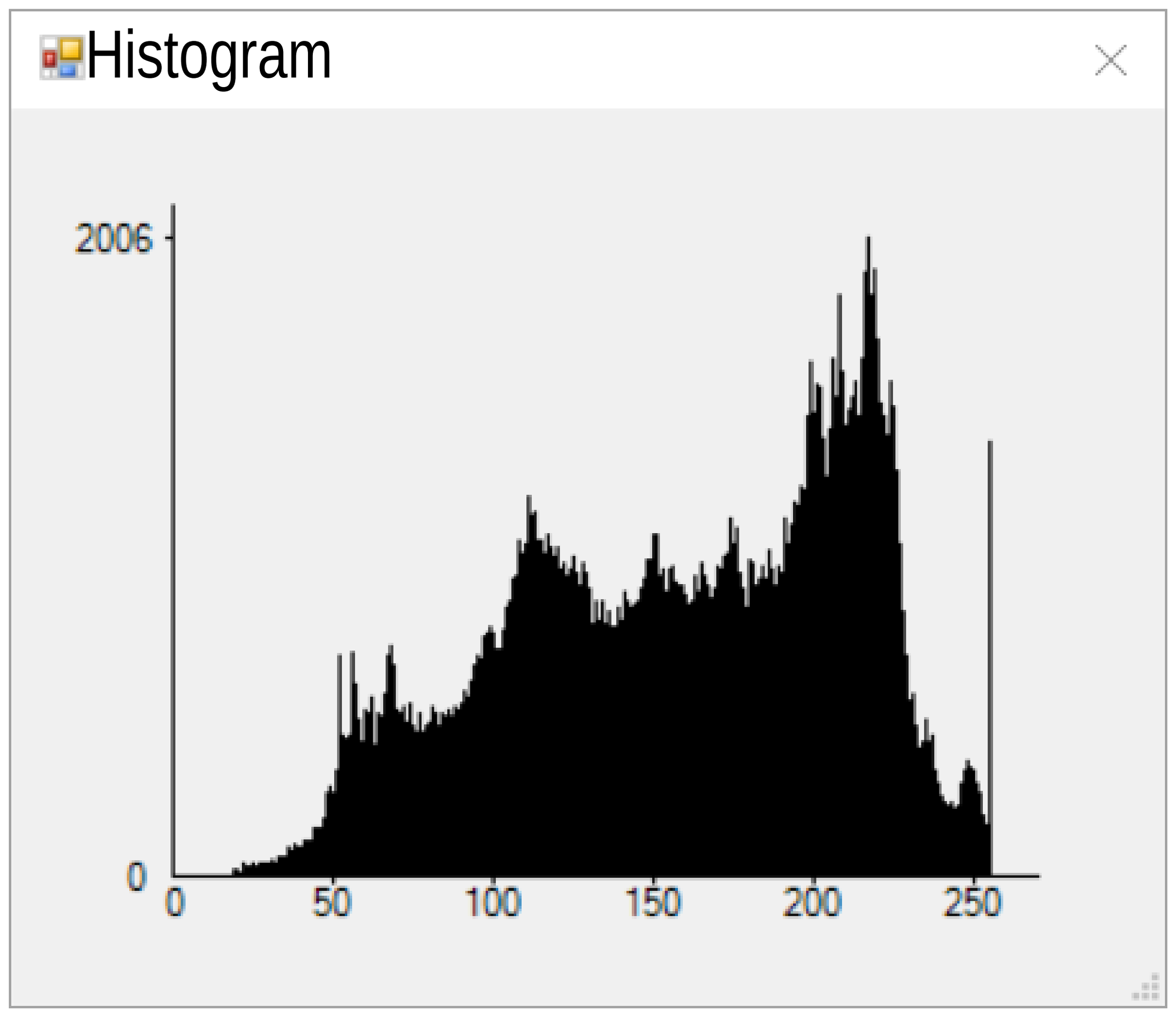

(g) Algorithm of this paper
