# Supplementary figures and images for: A 2D image 3D reconstruction function adaptive denoising algorithm
Source: PeerJ Comput Sci. 2023 Oct 3;9:e1604. doi: 10.7717/peerj-cs.1604 (PMC10557518; doi:10.7717/peerj-cs.1604)

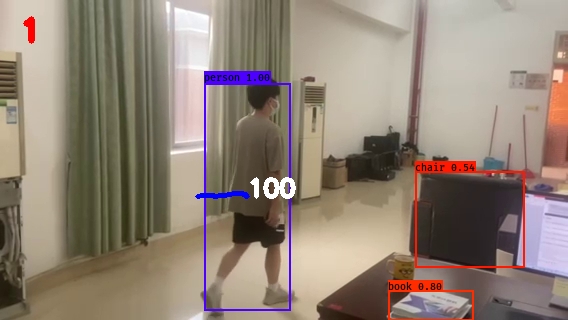

Supplement: Supplemental Information 2 [file peerj-cs-09-1604-s002.zip › cs-85591-data/2output/1 Target tracking.jpg]

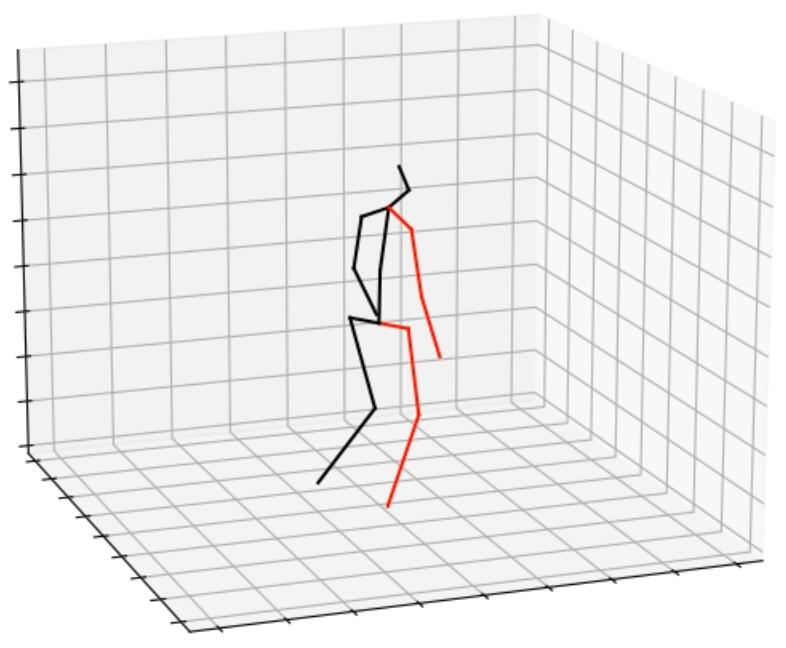

Supplement: Supplemental Information 2 [file peerj-cs-09-1604-s002.zip › cs-85591-data/2output/1-3D.jpg]

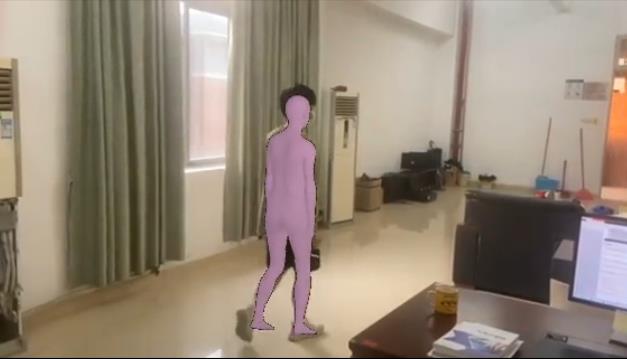

Supplement: Supplemental Information 2 [file peerj-cs-09-1604-s002.zip › cs-85591-data/2output/1-3D1.jpg]

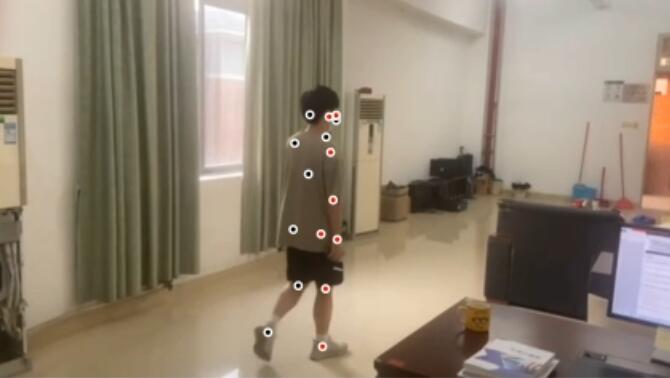

Supplement: Supplemental Information 2 [file peerj-cs-09-1604-s002.zip › cs-85591-data/2output/1.jpg]

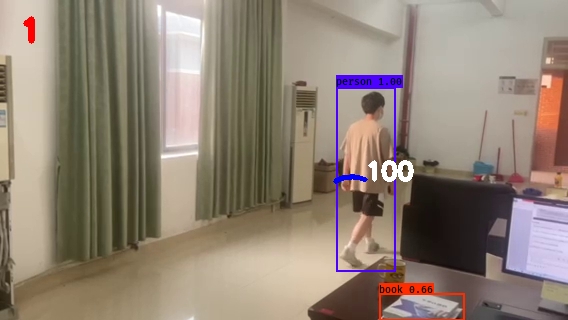

Supplement: Supplemental Information 2 [file peerj-cs-09-1604-s002.zip › cs-85591-data/2output/2 Target tracking.jpg]

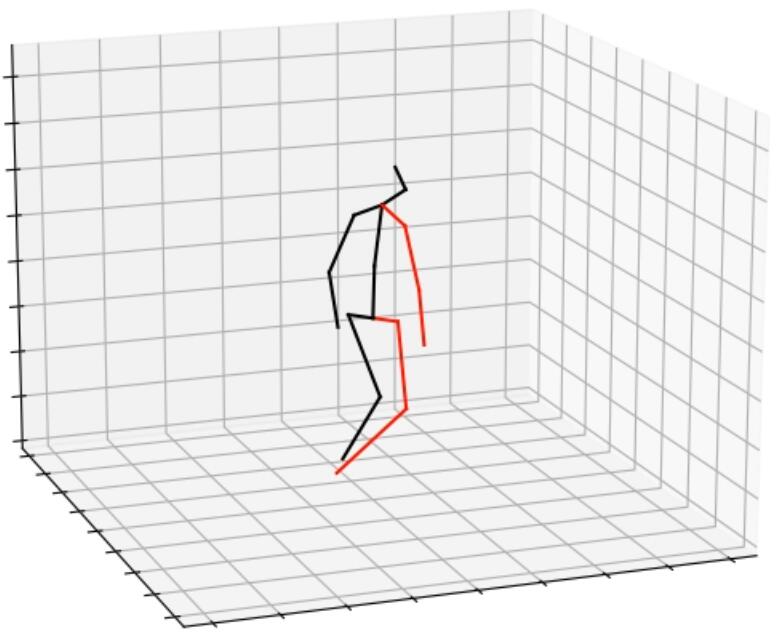

Supplement: Supplemental Information 2 [file peerj-cs-09-1604-s002.zip › cs-85591-data/2output/2-3D.jpg]

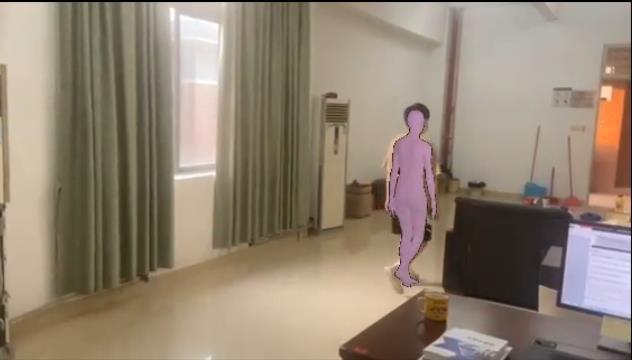

Supplement: Supplemental Information 2 [file peerj-cs-09-1604-s002.zip › cs-85591-data/2output/2-3D2.jpg]

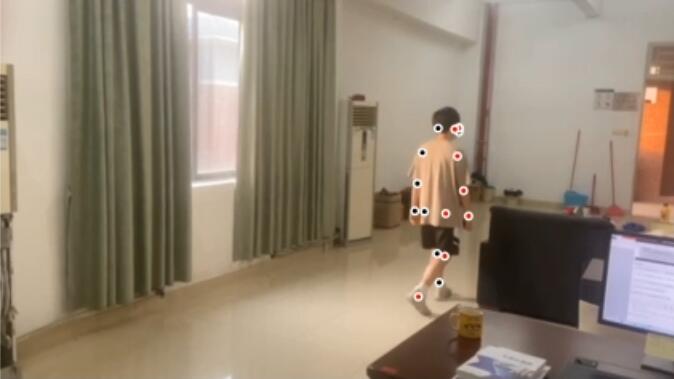

Supplement: Supplemental Information 2 [file peerj-cs-09-1604-s002.zip › cs-85591-data/2output/2.jpg]

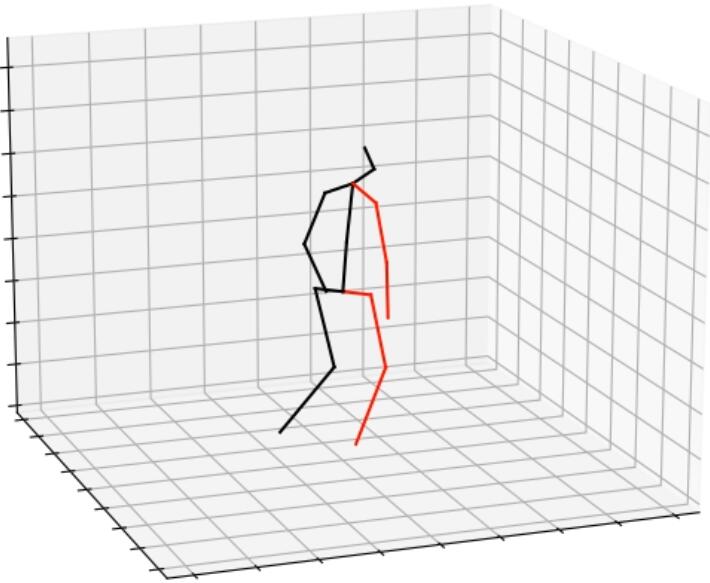

Supplement: Supplemental Information 2 [file peerj-cs-09-1604-s002.zip › cs-85591-data/2output/5-3D.jpg]

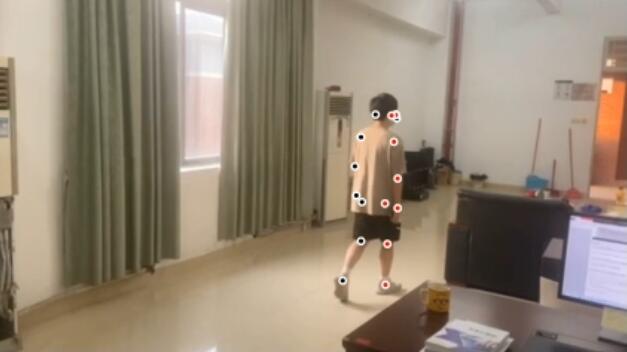

Supplement: Supplemental Information 2 [file peerj-cs-09-1604-s002.zip › cs-85591-data/2output/5.jpg]

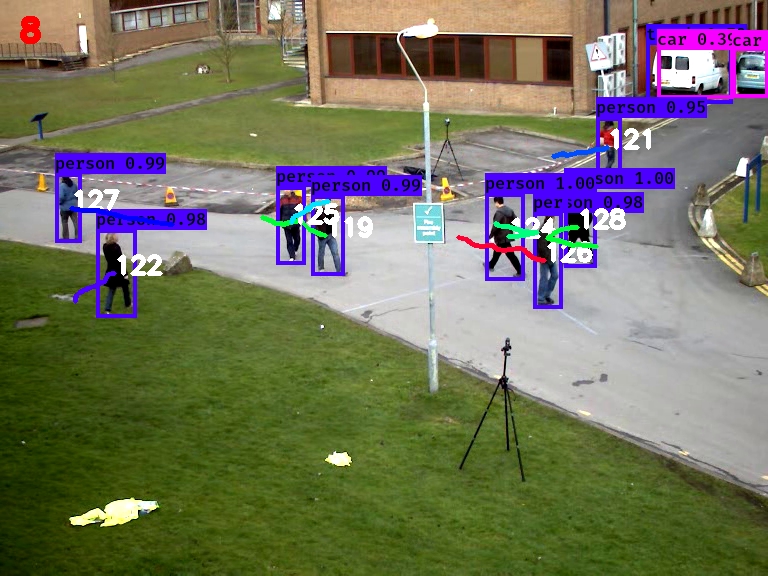

Supplement: Supplemental Information 2 [file peerj-cs-09-1604-s002.zip › cs-85591-data/2output/8.jpg]

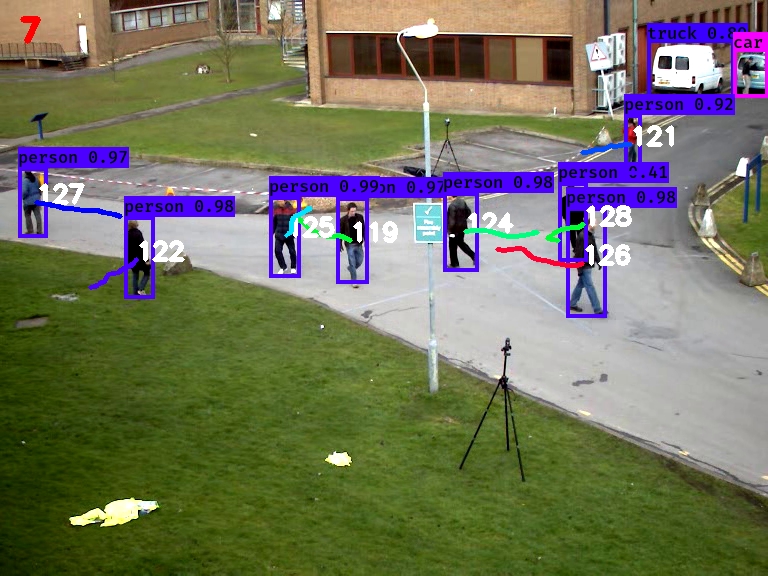

Supplement: Supplemental Information 2 [file peerj-cs-09-1604-s002.zip › cs-85591-data/2output/9.jpg]

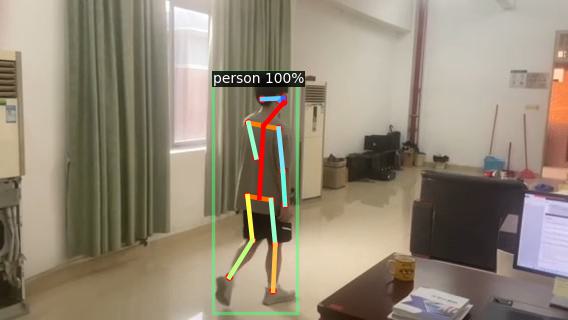

Supplement: Supplemental Information 2 [file peerj-cs-09-1604-s002.zip › cs-85591-data/2output/Key points (3).jpg]

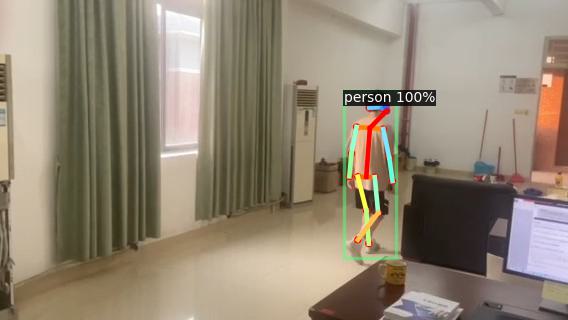

Supplement: Supplemental Information 2 [file peerj-cs-09-1604-s002.zip › cs-85591-data/2output/Key points (4).jpg]

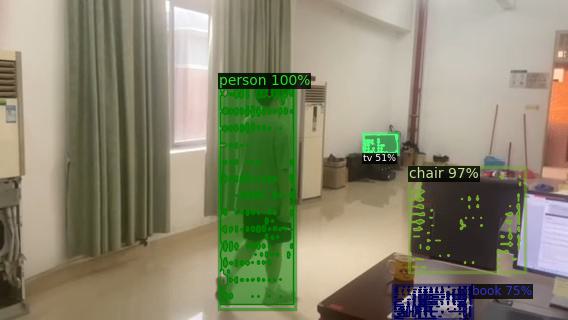

Supplement: Supplemental Information 2 [file peerj-cs-09-1604-s002.zip › cs-85591-data/2output/Panoramic effect (2).jpg]

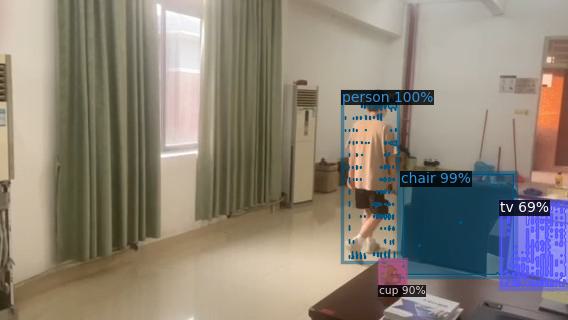

Supplement: Supplemental Information 2 [file peerj-cs-09-1604-s002.zip › cs-85591-data/2output/Panoramic effect (3).jpg]

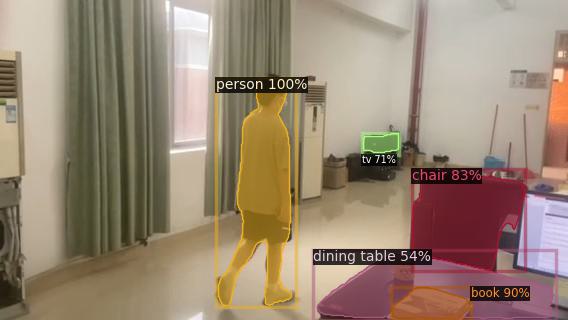

Supplement: Supplemental Information 2 [file peerj-cs-09-1604-s002.zip › cs-85591-data/2output/Splitting effect (1).jpg]

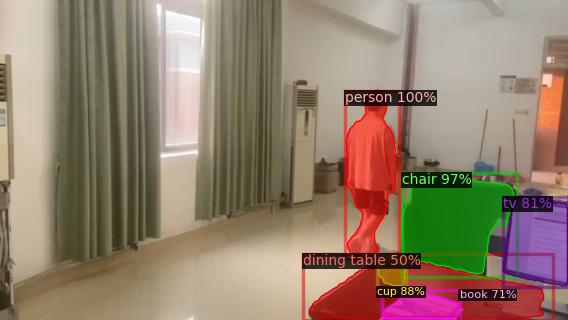

Supplement: Supplemental Information 2 [file peerj-cs-09-1604-s002.zip › cs-85591-data/2output/Splitting effect (2).jpg]

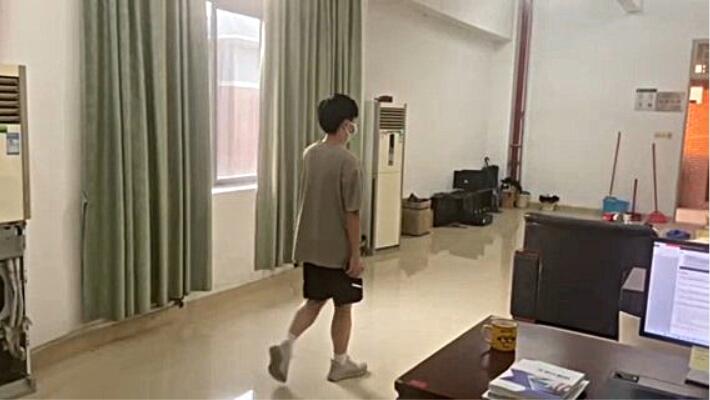

Supplement: Supplemental Information 2 [file peerj-cs-09-1604-s002.zip › cs-85591-data/Effects/1Algorithm for this paper.jpg]

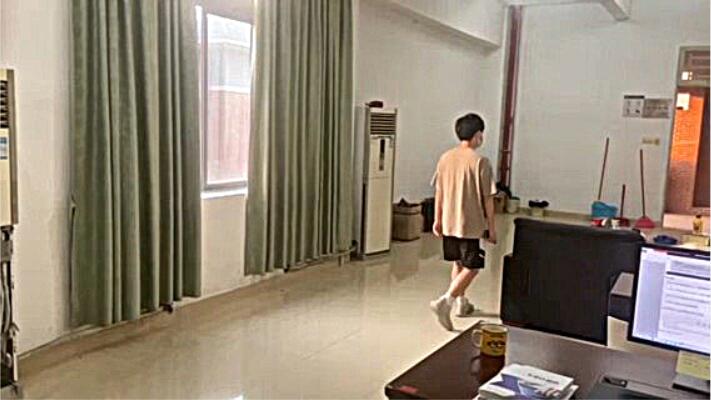

Supplement: Supplemental Information 2 [file peerj-cs-09-1604-s002.zip › cs-85591-data/Effects/2Algorithm for this paper.jpg]

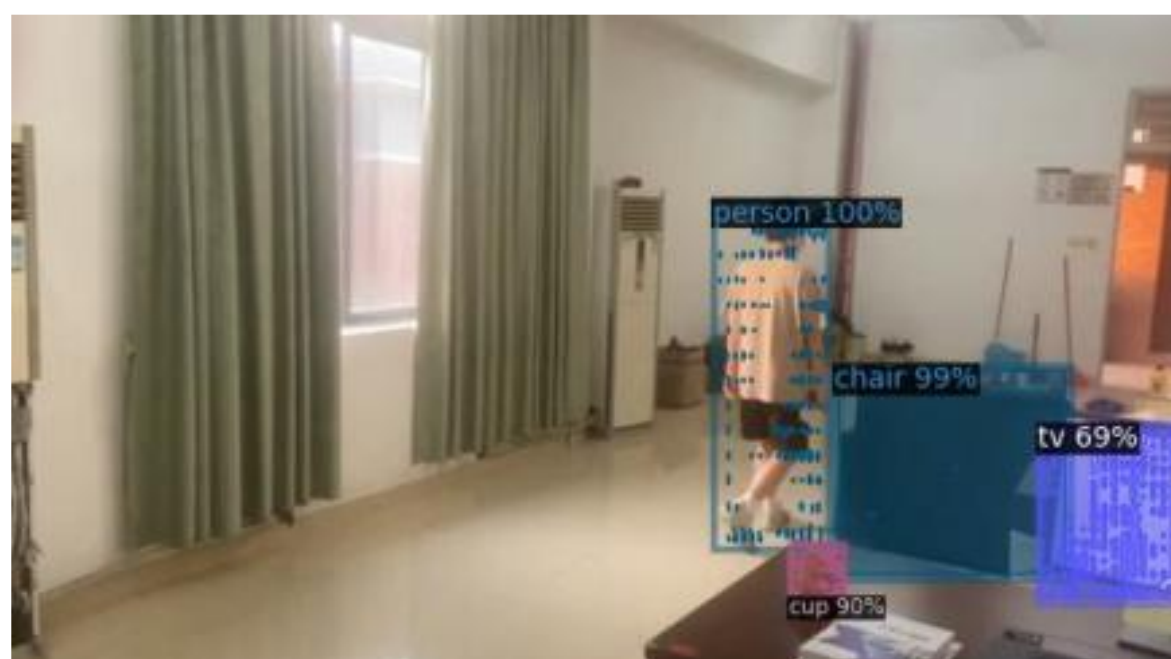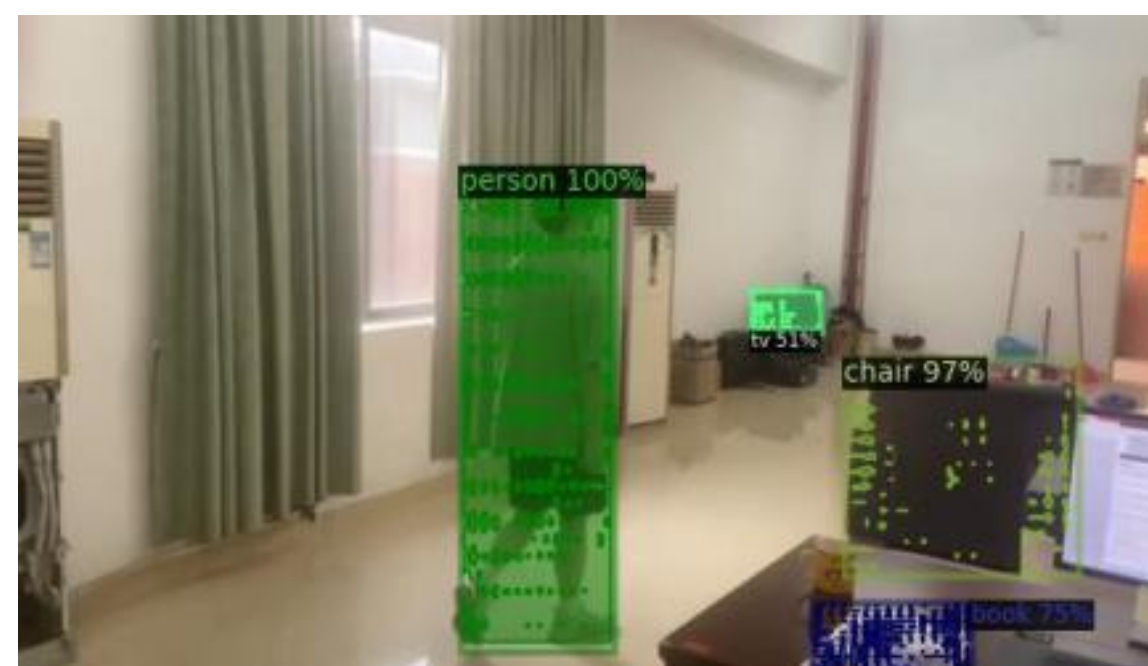

Supplement: Supplemental Information 2 [file peerj-cs-09-1604-s002.zip › cs-85591-data/Effects/Figure_1.pdf]

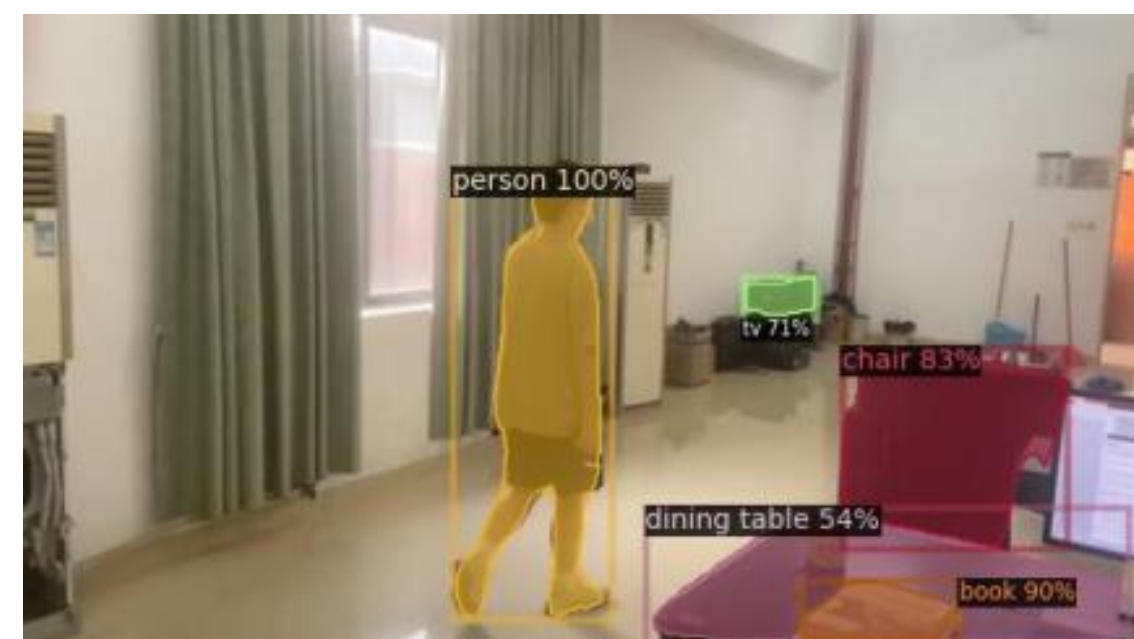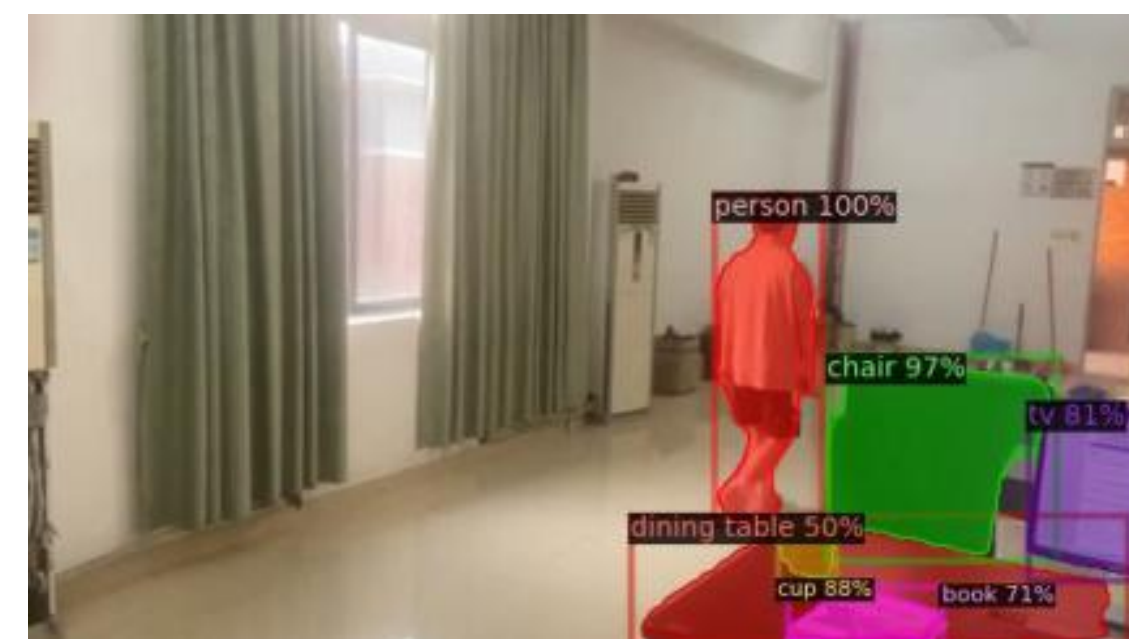

Supplement: Supplemental Information 2 [file peerj-cs-09-1604-s002.zip › cs-85591-data/Effects/Figure_2.pdf]

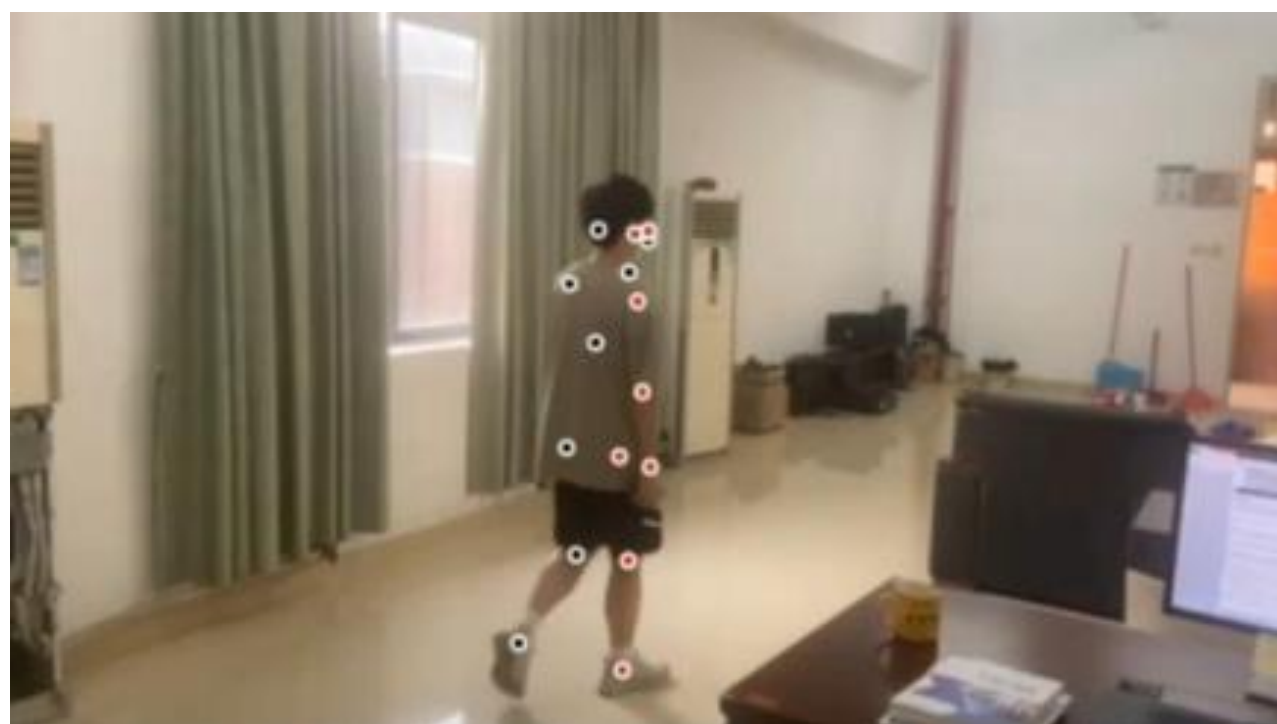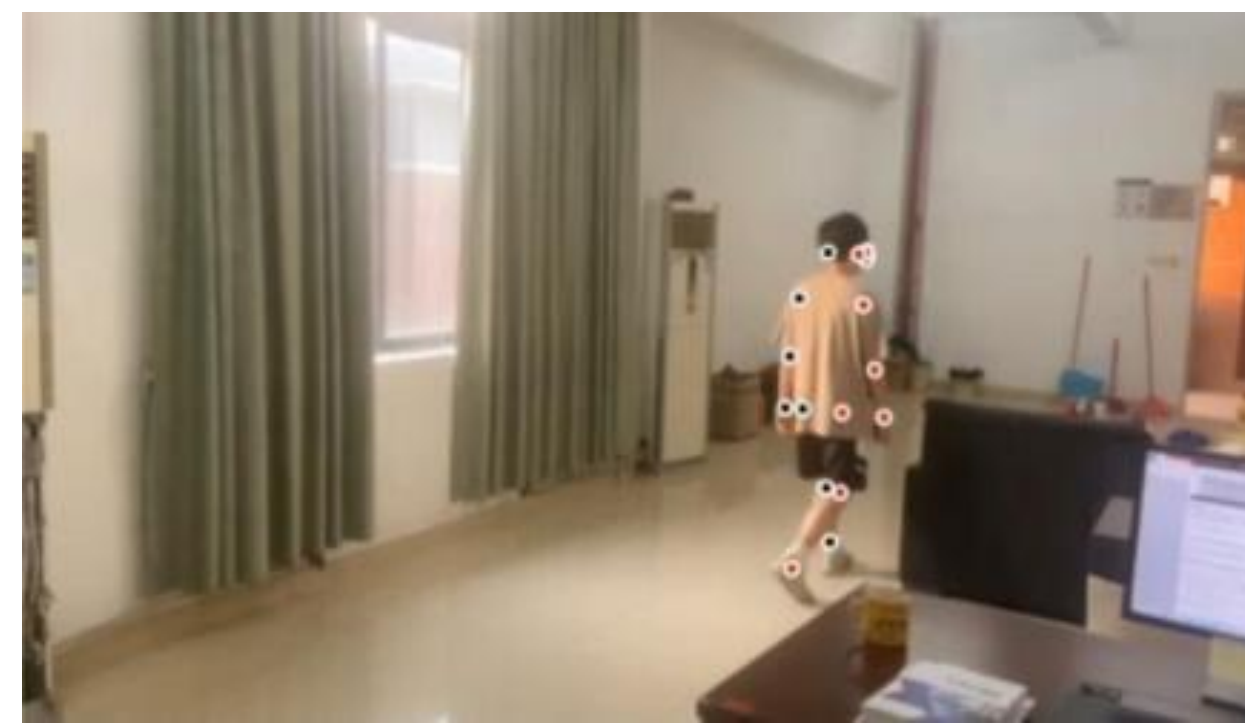

Supplement: Supplemental Information 2 [file peerj-cs-09-1604-s002.zip › cs-85591-data/Effects/Figure_3.pdf]

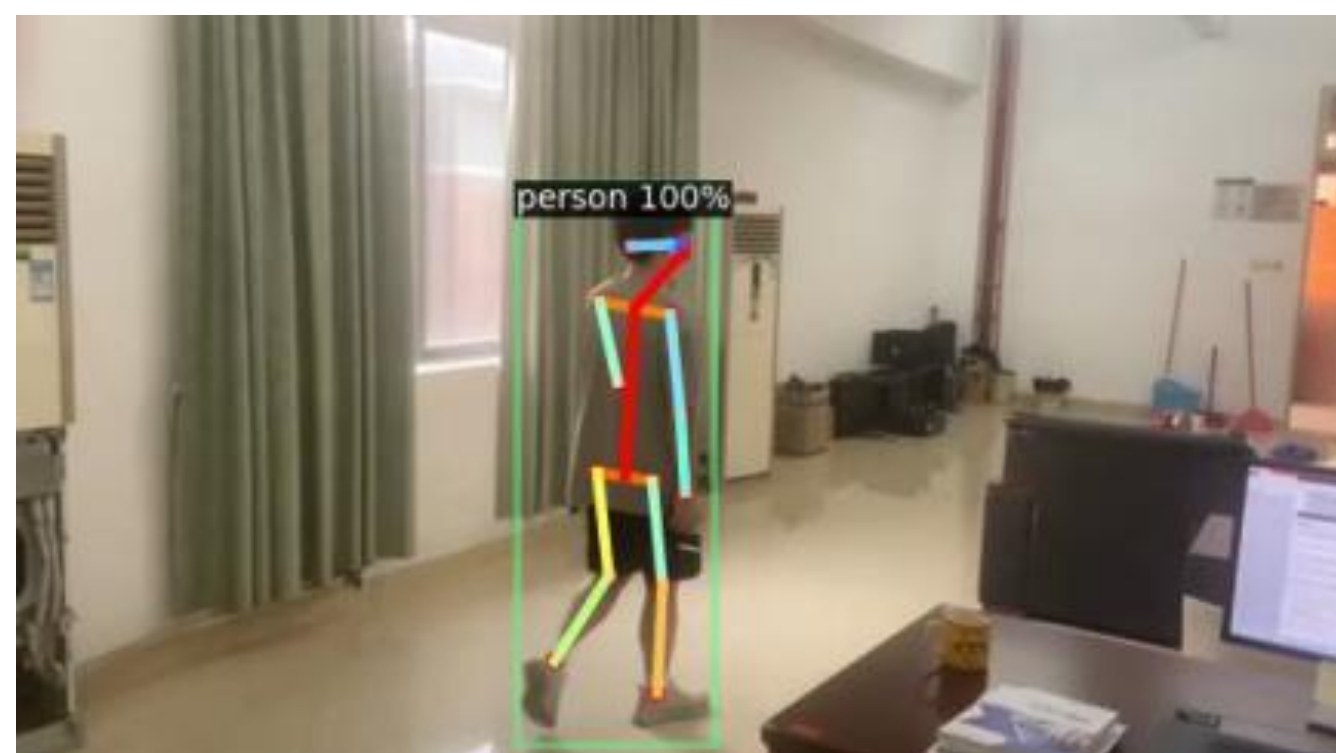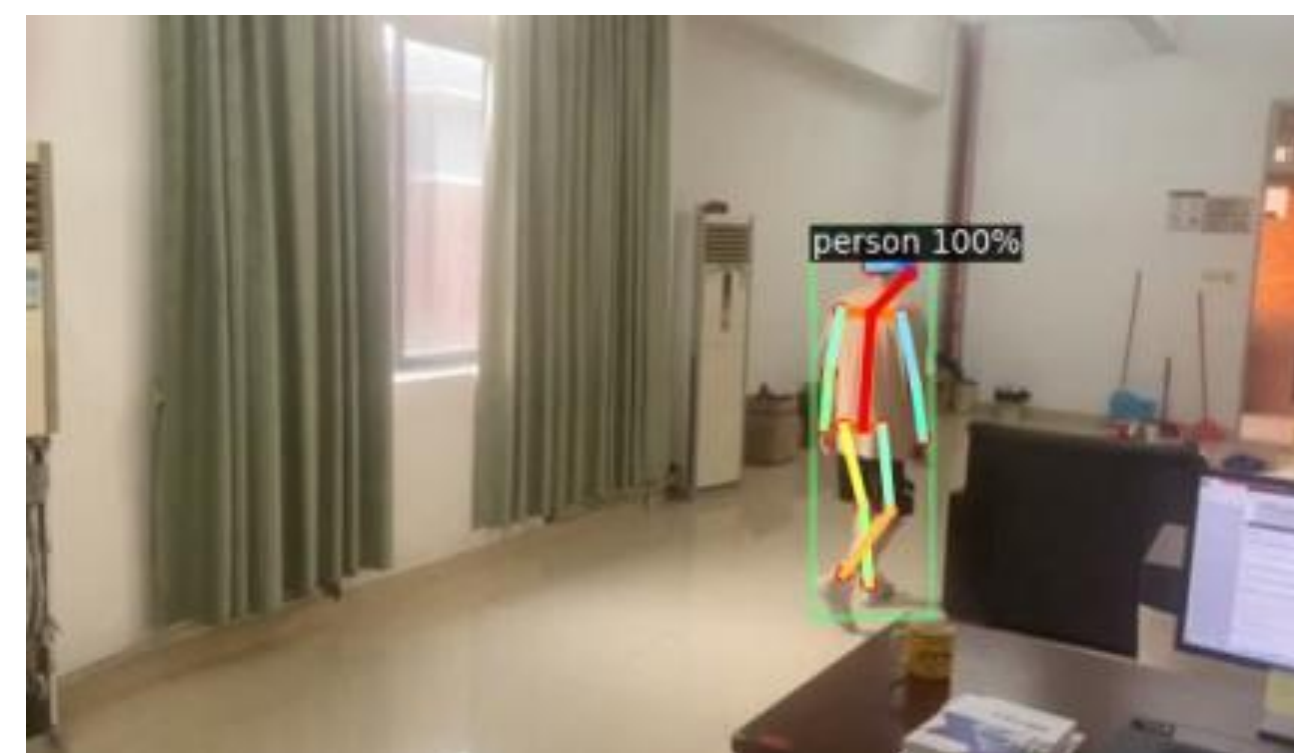

Supplement: Supplemental Information 2 [file peerj-cs-09-1604-s002.zip › cs-85591-data/Effects/Figure_4.pdf]

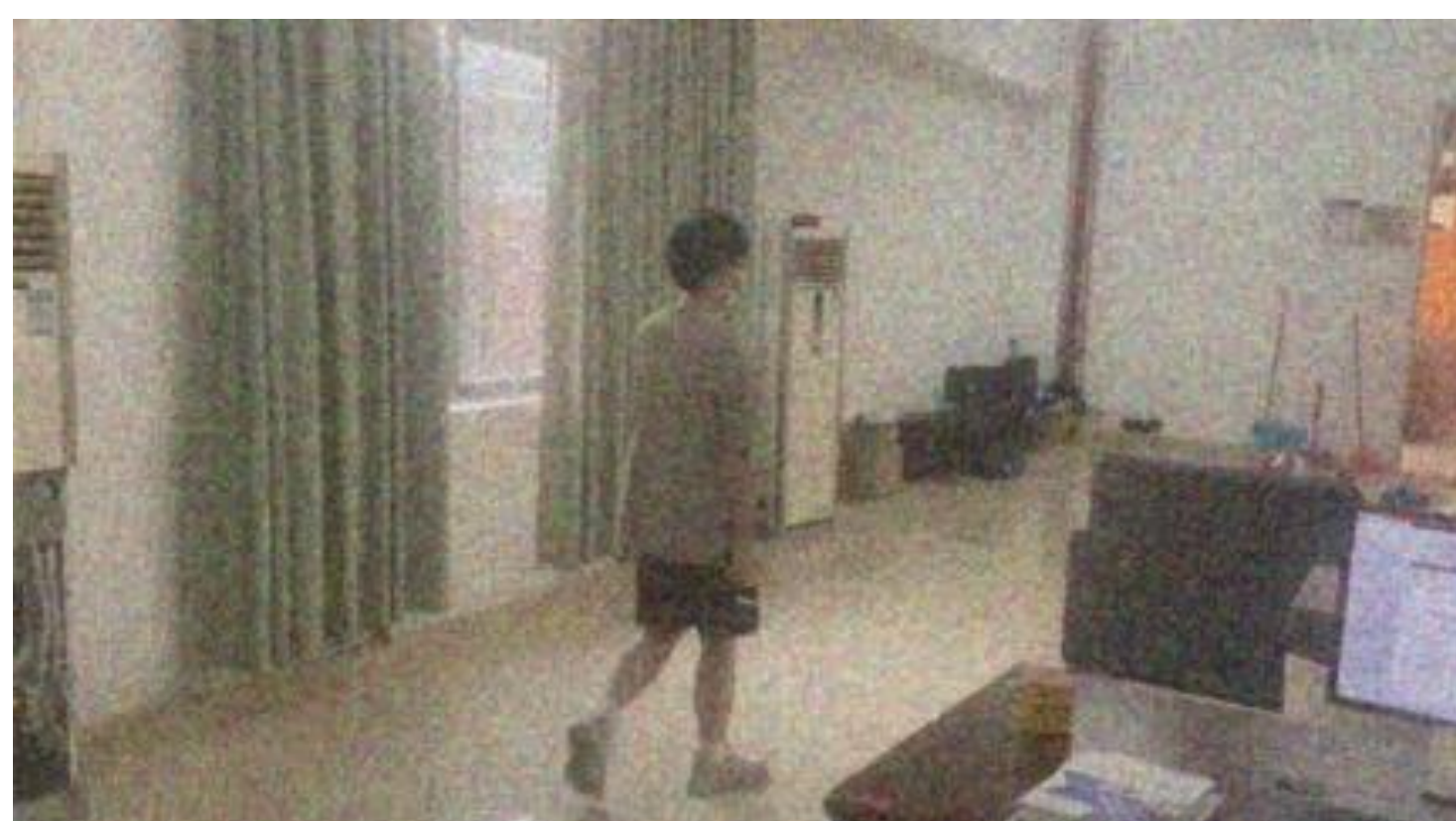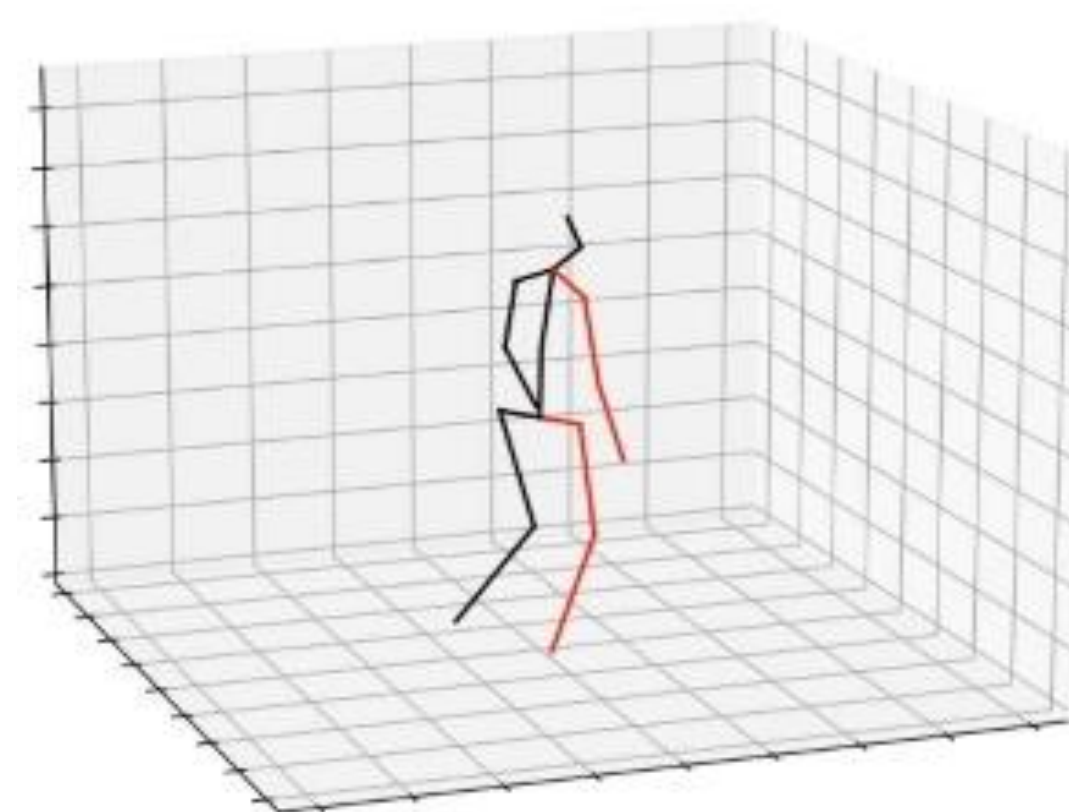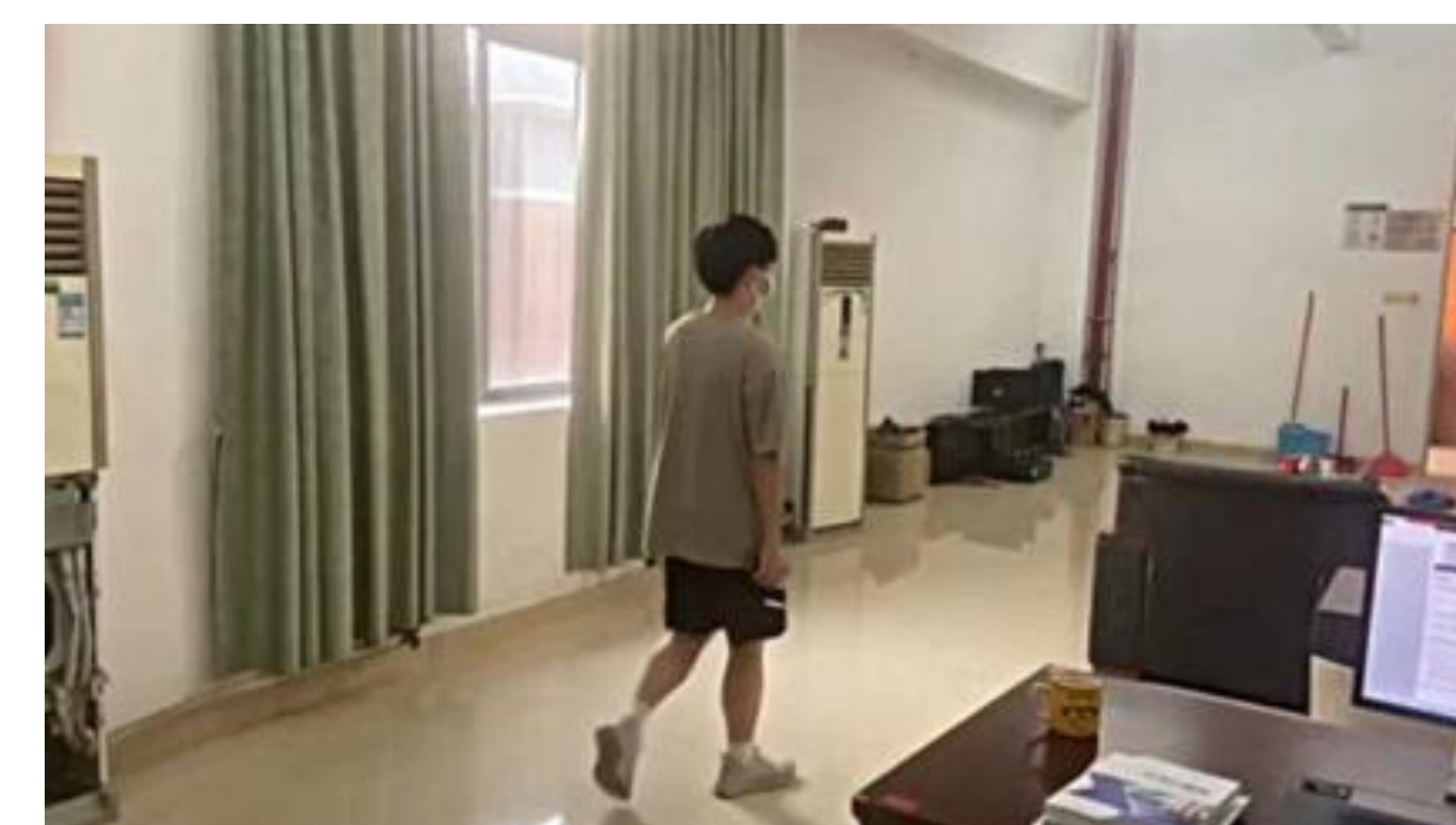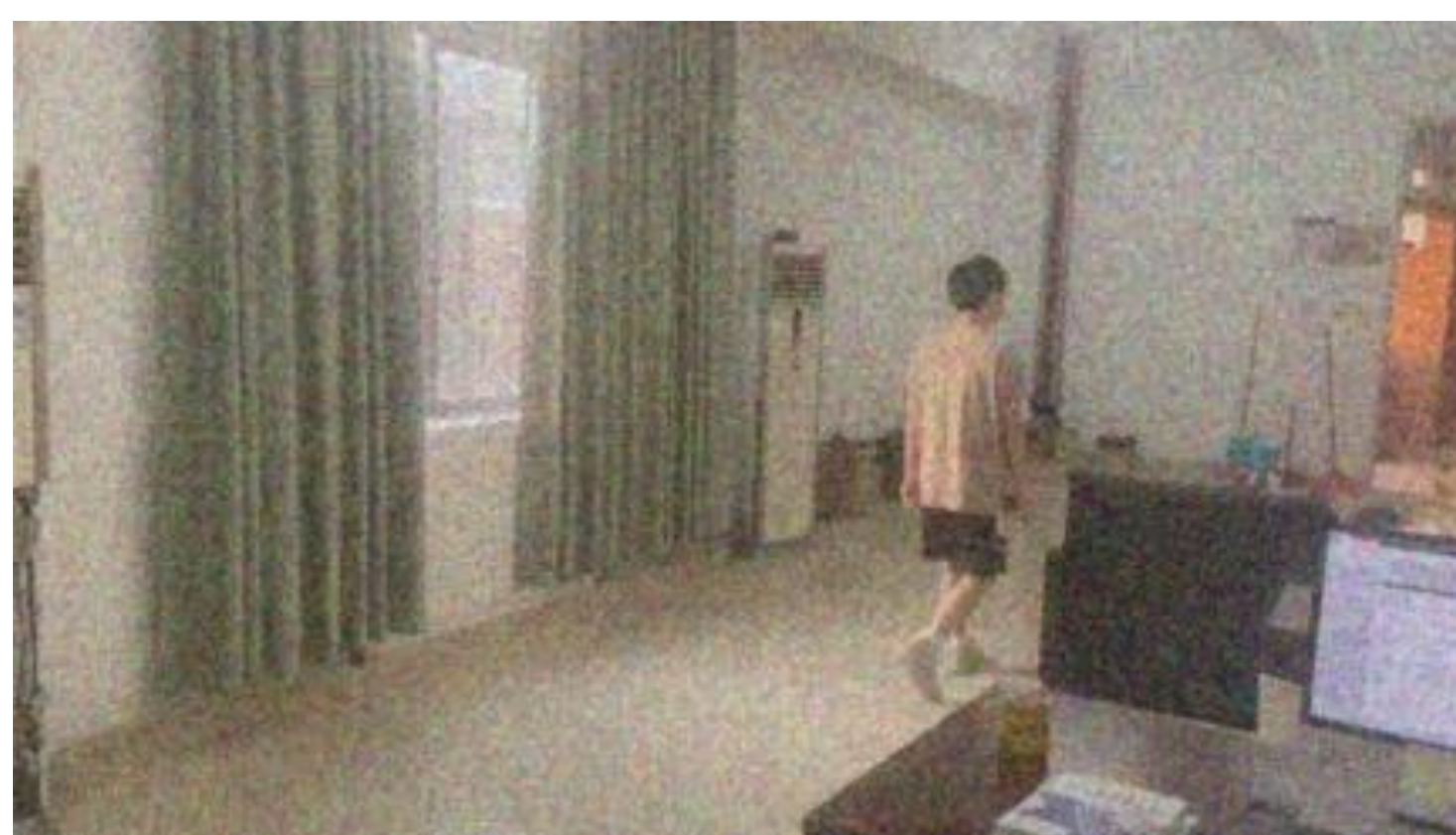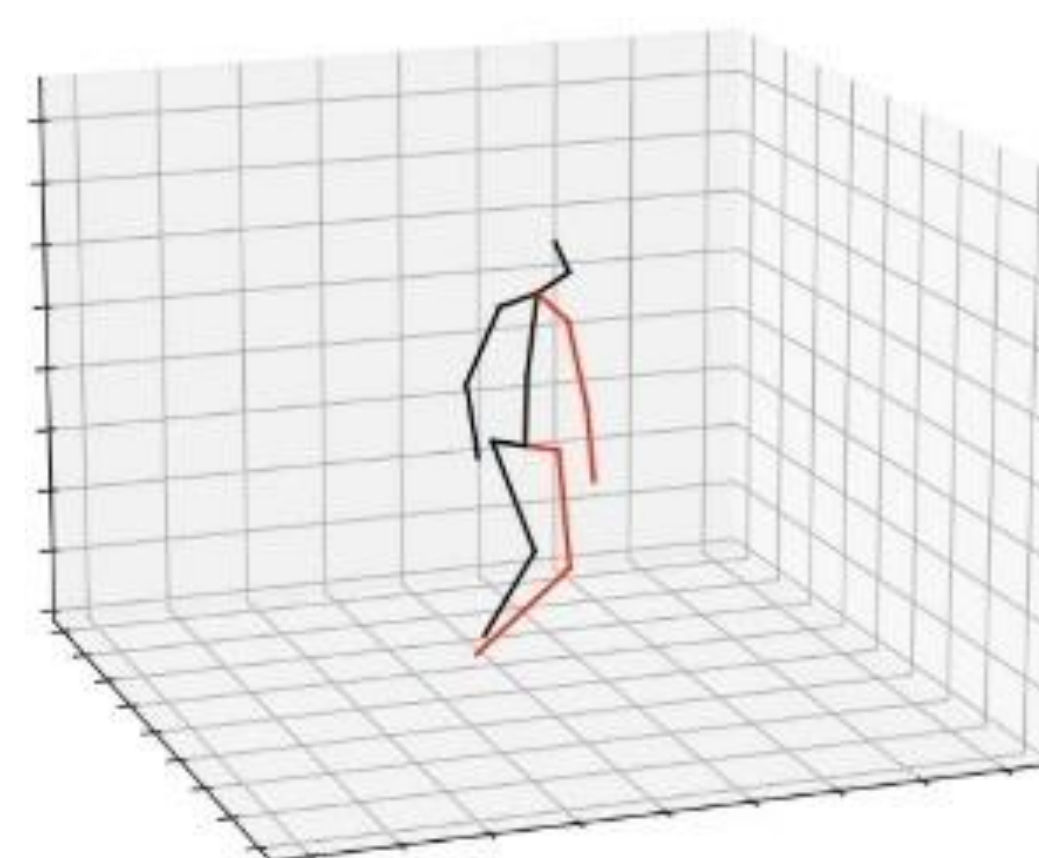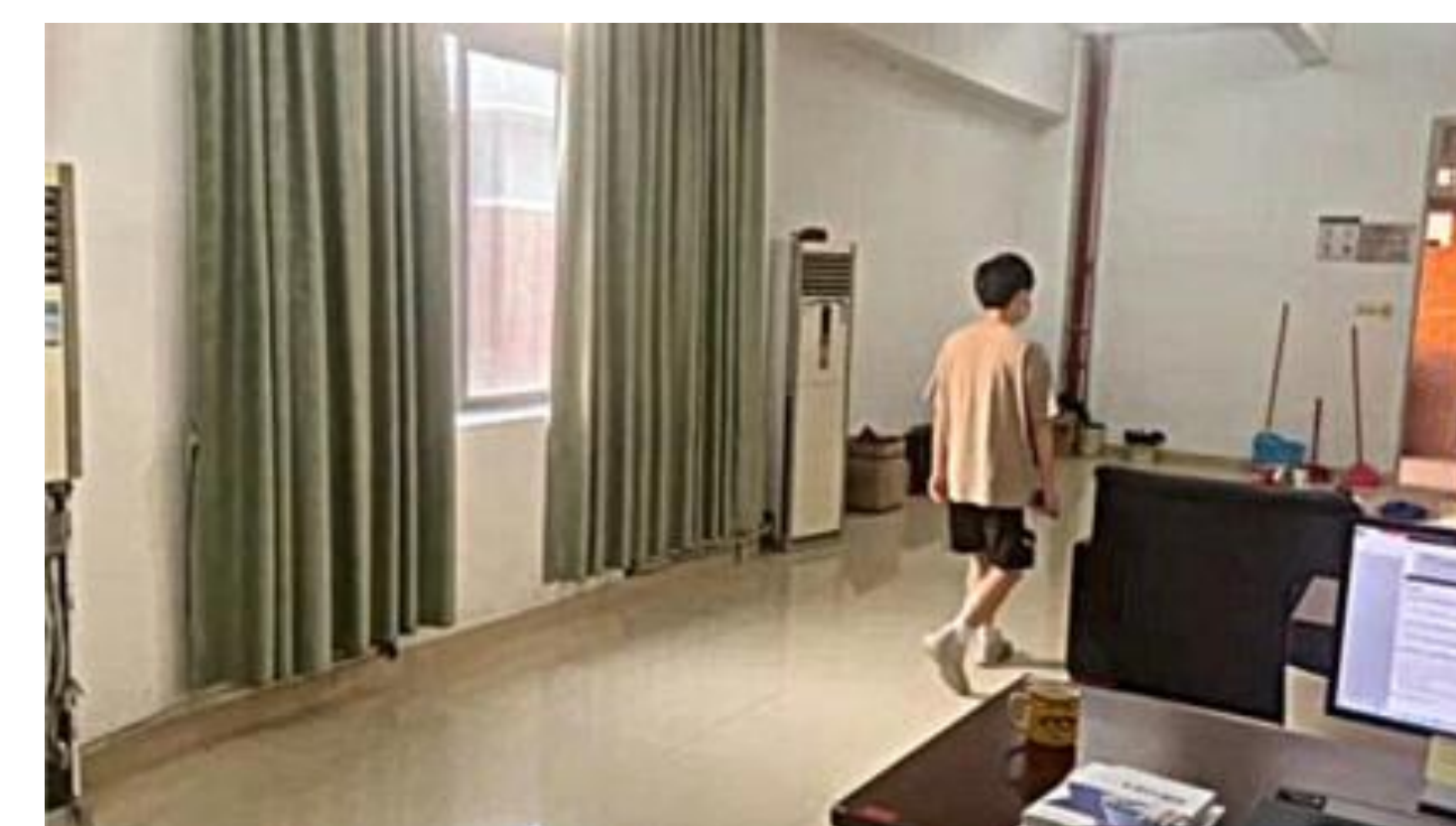

Supplement: Supplemental Information 2 [file peerj-cs-09-1604-s002.zip › cs-85591-data/Effects/Figure_5.pdf]

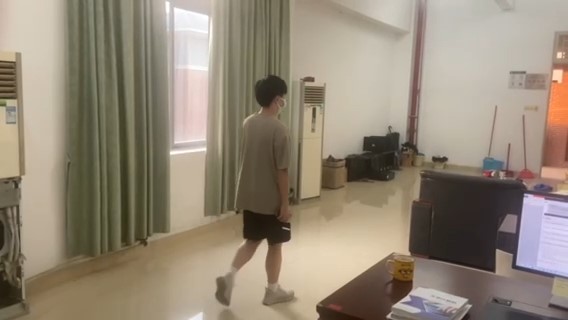

Supplement: Supplemental Information 2 [file peerj-cs-09-1604-s002.zip › cs-85591-data/Noisy images/1.jpg]

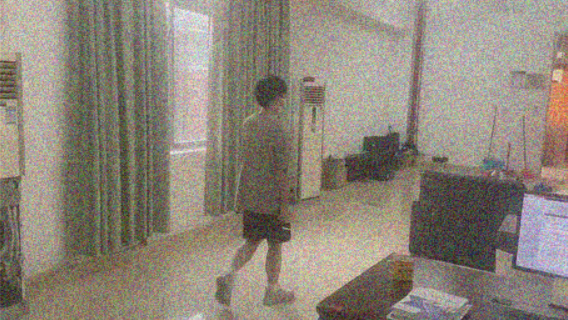

Supplement: Supplemental Information 2 [file peerj-cs-09-1604-s002.zip › cs-85591-data/Noisy images/1Noisy images.jpg]

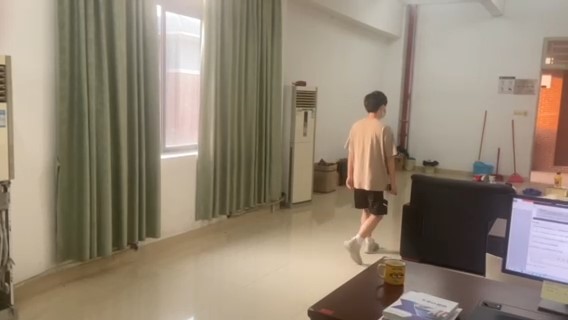

Supplement: Supplemental Information 2 [file peerj-cs-09-1604-s002.zip › cs-85591-data/Noisy images/2.jpg]

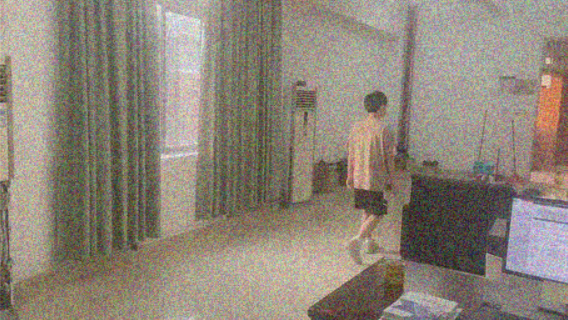

Supplement: Supplemental Information 2 [file peerj-cs-09-1604-s002.zip › cs-85591-data/Noisy images/2Noisy images.jpg]

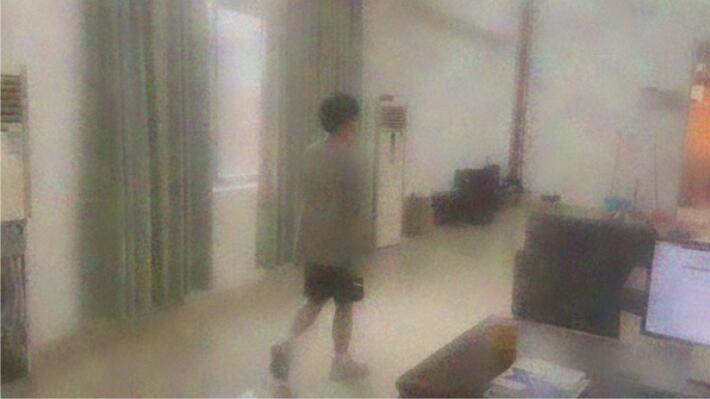

Supplement: Supplemental Information 2 [file peerj-cs-09-1604-s002.zip › cs-85591-data/Noisy images/Noisy images1.jpg]
